# Rapid acanthocytic schistocytosis and fulminant microangiopathic hemolysis as a preterminal event

**DOI:** 10.1002/ccr3.4247

**Published:** 2021-05-17

**Authors:** Habib Moshref Razavi, Richard Yu

**Affiliations:** ^1^ Division of Hematopathology Royal Columbian Hospital New Westminster BC Canada; ^2^ Department of Pathology and Laboratory Medicine University of British Columbia Vancouver BC Canada; ^3^ Division of Hematopathology Department of Laboratory Medicine Fraser Health Authority Surrey Memorial Hospital Surrey BC Canada

## Abstract

Isolated schistocytosis is a hematological emergency. Clinically significant schistocytosis requires 1% red cell fragments in a high power field. These include triangular fragments, crescents, helmet cells, keratocytes, or microspherocytes.

## 1 CASE DESCRIPTION

**FIGURE. 1:**
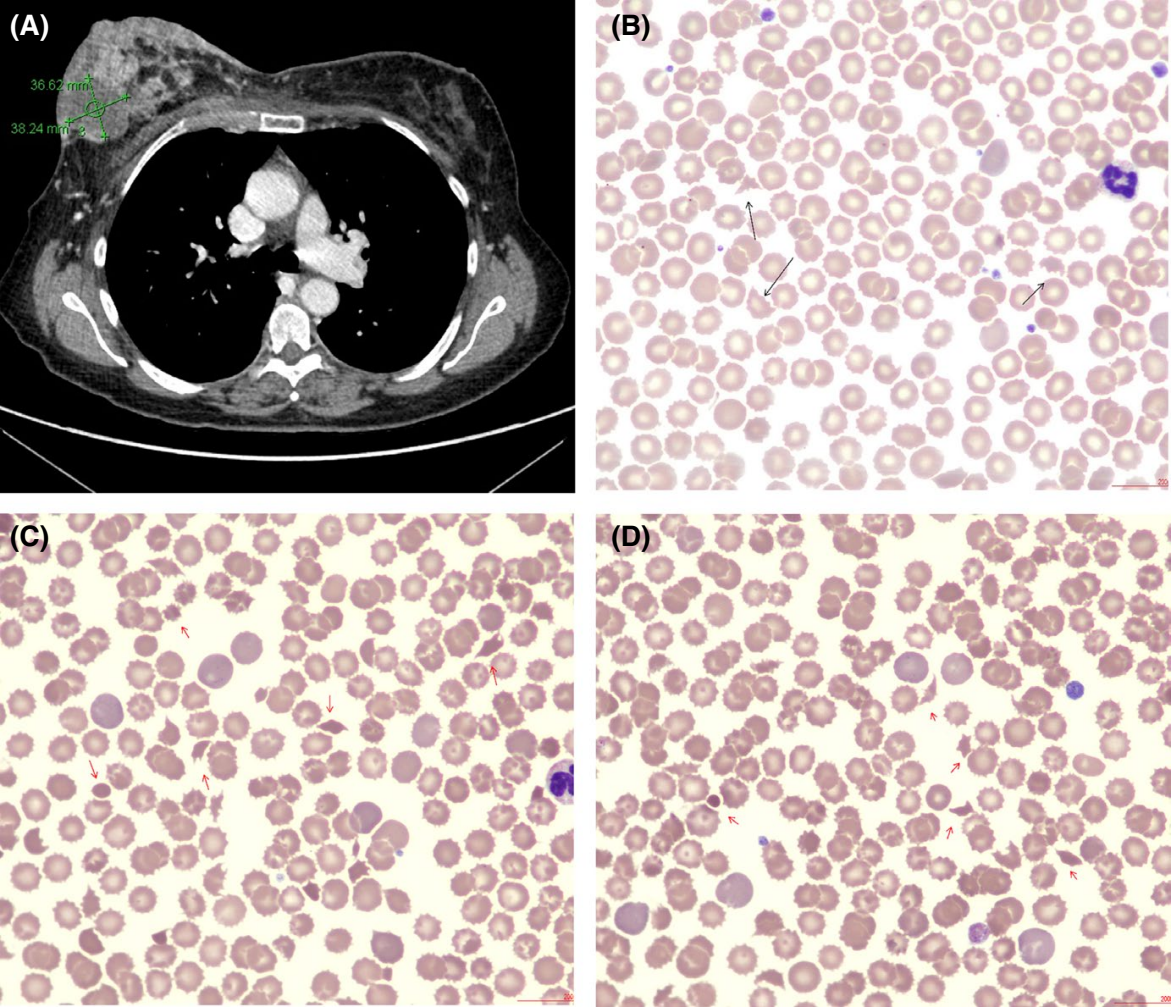
Morphological evidence of brisk cancer‐associated microangiopathic hemolysis is seen. (Panel A) shows the computed tomography image of a right‐sided ulcerating breast mass. At admission on day one (panel B), echinocytes, acanthocytes, and occasional schistocytes are seen. Over the next two days (panels C and D), significant red cell fragmentation developed just prior to patient's demise

A 59‐year‐old woman presented to our hospital with a right‐sided ulcerated breast mass (Figure 1, panel A). At admission, her CBC showed a white blood count of 17.9 × 10^9^/L, hemoglobin at 141 g/L, and a platelet count of 70 × 10^9^/L. Her reticulocyte count, lactate dehydrogenase, and unconjugated bilirubin were elevated (215 × 10^9^/L, 1479 U/L, and 209 µmol/L, respectively). Imaging showed multiple liver metastases, portal vein thrombosis, and possibly frank liver infarction. Her initial peripheral blood showed many echinocytes, occasional acanthocytes, and schistocytes (Figure 1, panel B, black arrows X 50 objective). Over the next two days along with a decreasing hemoglobin and progressive thrombocytopenia (84 g/L and 48 × 10^9^/L, respectively), a leucoerythroblastic picture, polychromasia, and significant schistocytosis developed in keeping with a brisk episode of microangiopathic hemolysis (Figure 1, panels C and D, respectively, X50 objective). Due to a fulminant hepatic failure, the patient shortly thereafter passed away. Possible causes of echinocytosis include storage and post‐transfusion artifacts, liver/renal disease, lactic acidosis, and hemolytic uremic syndrome. In addition to congenital causes (eg.,neuroacanthocytosis), acquired acanthocytosis is mainly due to liver disease. The International Council for Standardization in Hematology (ICSH) defines clinically significant schistocytosis as the presence of 1% red cell fragments seen in a high power field.^1^ Schistocytes and variants include triangular fragments, small crescents, helmet cells, keratocytes, or microspherocytes.

## AUTHOR CONTRIBUTIONS

HMR is the case pathologist. He prepared the manuscript and the figure. RY is the case pathologist. He reviewed and revised the manuscript.

## Data Availability

Data sharing not applicable—no new data generated.
